# A comparative analysis of the information content in long and short SAGE libraries

**DOI:** 10.1186/1471-2105-7-504

**Published:** 2006-11-16

**Authors:** Yi-Ju Li, Puting Xu, Xuejun Qin, Donald E Schmechel, Christine M Hulette, Jonathan L Haines, Margaret A Pericak-Vance, John R Gilbert

**Affiliations:** 1 Department of Medicine and Center for Human Genetics, Duke University Medical Center, Durham, North Carolina 27710, USA; 2 Center for Human Genetics Research Program, Vanderbilt University Medical Center, Nashville, Tennessee 37232, USA; 3 Department of medicine and Division of Neurology, Duke University Medical Center, Durham, NC 27710, USA

## Abstract

**Background:**

Serial Analysis of Gene Expression (SAGE) is a powerful tool to determine gene expression profiles. Two types of SAGE libraries, ShortSAGE and LongSAGE, are classified based on the length of the SAGE tag (10 vs. 17 basepairs). LongSAGE libraries are thought to be more useful than ShortSAGE libraries, but their information content has not been widely compared. To dissect the differences between these two types of libraries, we utilized four libraries (two LongSAGE and two ShortSAGE libraries) generated from the hippocampus of Alzheimer and control samples. In addition, we generated two additional short SAGE libraries, the truncated long SAGE libraries (tSAGE), from LongSAGE libraries by deleting seven 5' basepairs from each LongSAGE tag.

**Results:**

One problem that occurred in the SAGE study is that individual tags may have matched to multiple different genes – due to the short length of a tag. We found that the LongSAGE tag maps up to 15 UniGene clusters, while the ShortSAGE and tSAGE tags map up to 279 UniGene clusters. Both long and short SAGE libraries exhibit a large number of orphan tags (no gene information in UniGene), implying the limitation of the UniGene database. Among 100 orphan LongSAGE tags, the complete sequences (17 basepairs) of nine orphan tags match to 17 genomic sequences; four of the orphan tags match to a single genomic sequence. Our data show the potential to resolve 4–9% of orphan LongSAGE tags. Finally, among 400 tSAGE tags showing significant differential expression between AD and control, 79 tags (19.8%) were derived from multiple non-significant LongSAGE tags, implying the false positive results.

**Conclusion:**

Our data show that LongSAGE tags have high specificity in gene mapping compared to ShortSAGE tags. LongSAGE tags show an advantage over ShortSAGE in identifying novel genes by BLAST analysis. Most importantly, the chances of obtaining false positive results are higher for ShortSAGE than LongSAGE libraries due to their specificity in gene mapping. Therefore, it is recommended that the number of corresponding UniGene clusters (gene or ESTs) of a tag for prioritizing the significant results be considered.

## Background

Serial Analysis of Gene Expression (SAGE) introduced by Velculescu et al. [1] is a powerful open source method for profiling transcripts expressed in a given tissue. In this technique, mRNA transcripts are converted to cDNA and then processed 5' to the poly A+ tail to isolate short cDNA fragments called "tags." These tags are linked together into long concatemers and sequenced. The length of a SAGE tag is either 10 (short SAGE tag) or 17 (long SAGE tag) basepairs (bps) following a known restriction site. SAGE results are recorded as a list of distinct tags whose tag frequency can be tabulated to yield a quantitative measure of gene expression. The frequency counts of each SAGE tag reflect the abundance of the respective mRNA transcript expressed in the transcriptome of the tissue or cell type under study. Unlike microarray technology, which is limited to a finite number of known gene sequences arrayed on a chip, SAGE detects all transcripts expressed in a tissue sample and provides more quantitative information than microarrays. However, the disadvantages of SAGE are that the technique is expensive, time and labor intensive, and prone to sequencing errors [2]. Therefore, the total number of SAGE libraries produced for a study is generally smaller than a microarray study.

Annotation for a SAGE tag is a major task for SAGE data analysis. Many resources have been developed for mapping SAGE tags to genes, for instance, the SAGEmap from the National Center for Biotechnology Information (NCBI) [3] and the SAGE Genie from National Institutes of Health Cancer Genome Anatomy project [4]. Although these tools are useful, they rely on high quality databases to make confident tag-to-gene mapping. With only 14 bps (10 bps+ restriction sites) per a short SAGE (ShortSAGE) tag, it is impossible to directly screen a tag against the whole genome since 14 bps are insufficient to identify a unique genomic locus. UniGene Clusters  is the most frequently used database for searching corresponding transcriptome (e.g. genes or ESTs) of a SAGE tag. If a tag cannot be mapped to a UniGene cluster, it is impossible to determine if the tag is spurious (i.e. mis-sequenced, misincorporation of a nucleotide, not an mRNA), or represents a rare or novel gene not found in the UniGene databases. Therefore, it defeats the purpose of detecting unknown genes using SAGE tags. On the other hand, a LongSAGE tag (21 bps: 17 bps + restriction sites) is sufficiently long – making it possible to screen LongSAGE tags directly against the whole genome to identify its unique locus with a reasonable chance of success.

Due to the short length of a SAGE tag, it is common to see that a SAGE tag, especially the ShortSAGE tags, maps to multiple UniGene clusters which may be genes or ESTs,. When multiple genes or ESTs are found for a single tag, it is impossible to differentiate the tag count for genes/ESTs that have the same SAGE tag sequence. Therefore, when such ShortSAGE tag is found to express differentially between two samples, it cannot be determined which gene(s) or EST(s) is expressing differentially. This can lead to serious problems in interpreting gene expression levels between different tissues or states. The longer tags from the LongSAGE libraries may help correct this problem in addition to providing the opportunity to identify new and unique genes.

Although LongSAGE libraries possess several inherent advantages vis-à-vis ShortSAGE libraries, to date, available studies that compared the information content of ShortSAGE and LongSAGE are limited [2,5]. In addition, previous studies focused more on the tag annotation issue than other topics. Lu et al. generated four LongSAGE libraries using colon cell lines with/without a p53 mutation under either normal oxygen or hypoxia conditions. Based on these four LongSAGE libraries, they generated four ShortSAGE libraries by extracting the 10-bp tags from the longSAGE tags. They limited their analyses on the confident tags, that is, the tags with counts > 1. They concluded that the ShortSAGE more efficiently identifies differentially expressed genes than LongSAGE. They also found that only 4–7% of the redundant confident ShortSAGE tags can be resolved by confident LongSAGE tags. Similarly, van Ruissen et al. [2] did not find improvement on SAGE tag annotation by LongSAGE tag. That is, both ShortSAGE and LongSAGE have about 30% of tags with reliable annotation. Overall, these studies seem to favor ShortSAGE libraries.

In this study, we investigated various issues related to the information content of LongSAGE and ShortSAGE libraries. Different from Lu et al. [5], we utilized two types of ShortSAGE libraries. One is modified from the LongSAGE libraries as Lu et al. did. The other is the real ShortSAGE library sequenced from the samples. We generated four SAGE libraries (Two LongSAGE and two ShortSAGE) using human brain tissue samples of two Alzheimer cases and two controls. We attempted to address the following: (1) determine the number of tags that can be matched to UniGene Clusters using LongSAGE and ShortSAGE tags; (2) evaluate tags that we were unable to assign to UniGene Clusters; (3) compare the number of significant differentially expressed genes that can be derived from LongSAGE and ShortSAGE libraries; and (4) investigate the use and potential advantages of LongSAGE tags in identifying novel genes not listed in UniGene database.

## Results

Table 1 summarizes the basic tag information for each SAGE library. More than 70,000 tags were extracted from both LongSAGE and ShortSAGE libraries. The number of tag counts per tag ranges from one to 2,202 for long SAGE tags, and one to 1,098 for short SAGE tags. Interestingly, the total tag counts and the numbers of distinct tags (unique tags) were higher in AD than control samples in both LongSAGE and ShortSAGE libraries. For instance, there are 34,475 unique tags in L_AD and 30,581 in L_Ctrl, indicating more tags expressed in the AD than control tissues. Since not all tags are expressed in both libraries of AD and control samples, the number of tags that are expressed in at least one of libraries increases to 55,093 for LongSAGE, 43,937 for tSAGE, and 37,900 for ShortSAGE compared datasets. Furthermore, the overall frequency of SAGE tags mapped to UniGene build 182 for each library is not very high. For instance, we found 14,643 tags (42.5%) in L_AD and 11,646 tags (38.1%) in L_Ctrl that map to the UniGene database, which lead to a large number of orphan tags (no UniGene IDs) in each library (Table 1).

**Table 1:** Summary of SAGE tags for four SAGE libraries

Libraries*	**L_AD**	**L_Ctrl**	**S_AD**	**S_Ctrl**
	(T_AD)**	**(T_Ctrl)**		
**Unique tags**	34,475 (28,804)	30,581 (25,145)	25,140	23,126
**No. total tag counts**	80,292	75,018	78,126	70,456
**No. SAGE tags**	14,643 (24,129)	11,646 (20,640)	21,367	19,787
**mapped to UNIGENE**				
**No. orphan tags**	19,832(4,675)	18,935(4,505)	3,773	3,339

*Library abbreviation is as the following: LongSAGE AD (L_AD), tSAGE AD (T_AD), LongSAGE control (L_Ctrl), tSAGE control (T_Ctrl), ShortSAGE AD (S_AD); ShortSAGE control (S_Ctrl).

**The information of truncated long SAGE (tSAGE) libraries for AD and control are listed in the parenthesis.

Applying the same strategy described in Lu et al. [5], we evaluated the tag-to-gene relationship using confident LongSAGE tags, which are defined for the tags with counts > 1. Under this constraint, we still observed more LongSAGE tags in L_AD than L_Ctrl. Interestingly, we observed similar frequencies of redundant short tags. We found that only about 4.9 – 5.7% of tSAGE tags mapped to multiple LongSAGE tags (Table 2). Further, more than 70% of confident tags can be mapped to UniGene Cluster(s), indicating that the overall low tag-to-gene mapping for each library is mainly coming from those tags with tag counts < 2 (non-confident tags).

**Table 2:** Redundancy and tag-to-gene mapping for unique tags with tag counts > 1 (confident tags).

**Library**	**Unique tags with counts > 1 (Confident tags)**	**Redundant tSAGE tags^b^**	**Confident tags mapped to UniGene**
**L_AD**	8,670	---	6,547 (75.5%)
**L_Ctrl**	7,210	---	5,149 (71.4%)
**T_AD**	7,699	379 (4.9%)	7,265 (94.4%)
**T_Ctrl**	6,195	356 (5.7%)	5,762 (93.0%)

^a^Redundant tags refer to tSAGE tags matching to more than one LongSAGE tag with counts greater than 1.

**Figure 1:**
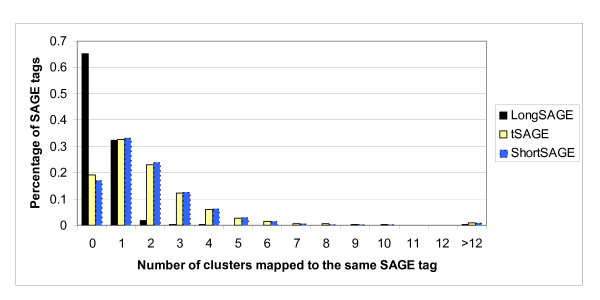
**Distribution of SAGE tags**. The distribution of SAGE tags depicted by the number of corresponding clusters in the LongSAGE, truncated LongSAGE, and short SAGE datasets.

As expected, the tag-gene relationship is more specific for the LongSAGE tags than the short SAGE tags. Figure 1 depicts the distribution of tags based on the number of their corresponding UniGene clusters for each compared dataset. The LongSAGE library shows a large percentage of orphan tags (65%) in comparison to tSAGE and ShortSAGE that have about 18% of orphan tags. This is expected, as the probability of mapping to a UniGene Cluster is much smaller for a long SAGE tag due to the extra seven bps. Three compared libraries show a similar percentage of tags mapping to a single UniGene cluster, that is, 32.3% for the LongSAGE, 32.7% for the tSAGE, and 33.1% for the ShortSAGE libraries. However, 97.3% of LongSAGE tags are either orphan tags or map to a single UniGene cluster, while both tSAGE and ShortSAGE libraries still have about 50% of tags mapping to more than one UniGene clusters. The maximum number of UniGene clusters that correspond to a single tag was 15 for the LongSAGE tags, and 279 for both tSAGE and ShortSAGE tags. This may imply that there is a higher chance of obtaining false matches for a ShortSAGE tag than a LongSAGE tag. For instance, of the 17,793 LongSAGE tags that map to a single UniGene cluster, only 5,749 tags map to a single UniGene cluster after converting to the tSAGE tags, and the rest contribute to the pool of tags that map to more than one cluster which may represent false matches. As theorized, the increased specificity in gene mapping offered by the LongSAGE tags is substantial, compared to ShortSAGE tags.

**Figure 2:**
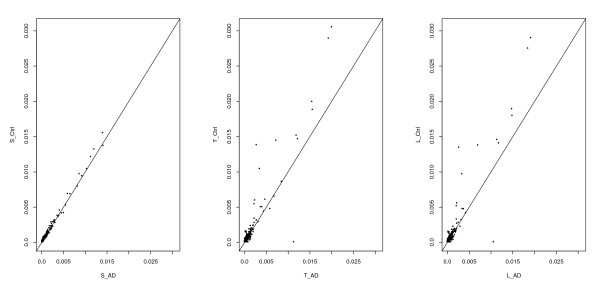
**Tag frequency comparison**. Comparisons of tag frequencies between AD and controls of LongSAGE, ShortSAGE, and tSAGE libraries.

When we compared the expression pattern between AD and control for three types of libraries: LongSAGE, tSAGE, and ShortSAGE, both LongSAGE and tSAGE libraries share strong similarity (Figure 2). This is reasonable as they were based on the same samples. Unexpectedly, S_AD and S_Ctrl show very similar expression levels for the majority of genes, which is different from the case and control samples used for LongSAGE and tSAGE libraries. Our testing results reflected the expression patterns in Figure 2. We detected 380 LongSAGE tags, 400 tSAGE tags, and 156 ShortSAGE tags with significant differential expression between AD and control (P < 0.05). Clearly, we detected fewer tags in the ShortSAGE dataset than the other two. Although significant, this difference could be due to gene expression variation between samples with the same disease status.

**Figure 3:**
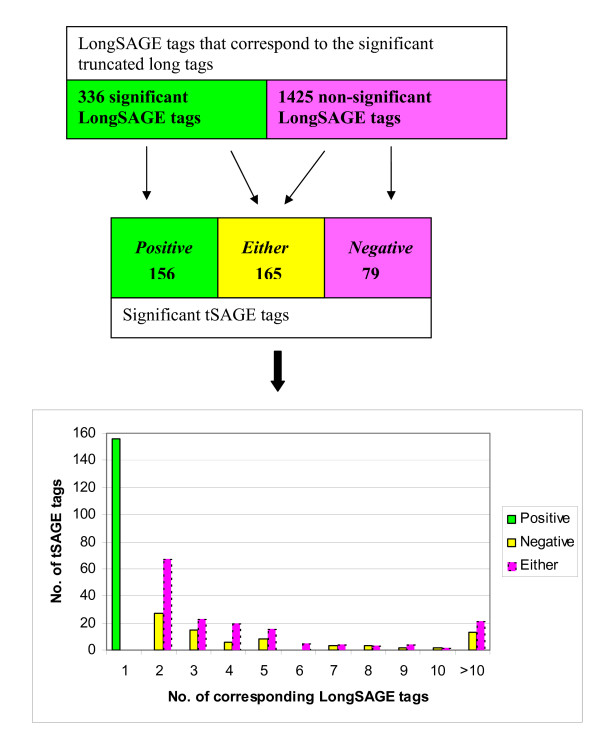
**The property of significantly differentially expressed tSAGE tags**. A diagram to relate the LongSAGE tags to 400 tSAGE tags that are significantly differentially expressed between AD and control. The distribution of the tSAGE tags is summarized based on the number of their corresponding LongSAGE tags.

Since both LongSAGE and tSAGE libraries were derived from the same samples, we used these two datasets to measure the relative ability of long and short SAGE libraries to detect altered gene expression. We found that the 400 significant differentially expressed tSAGE tags were derived from 336 significant and 1,425 non-significant LongSAGE tags. We assigned each tSAGE tag to one of three categories that are defined based on the testing results of its corresponding long tags: (1) *Positive* group, if all corresponding LongSAGE tags for the tSAGE tag are significant; (2) *Negative* group, if all corresponding LongSAGE tags for the tSAGE tag are not significant; or (3) *Either *group, if the corresponding LongSAGE tags for the tSAGE tag are a combination of significant and non-significant. Figure 3 depicts the relationship between the 400 significant tSAGE tags and their corresponding LongSAGE tags in these three groups. The 400 tSAGE tags distributed as 156 tSAGE tags in the *Positive *group, 79 in *Negative *group, and 165 in the *Either *group. Interestingly, each tSAGE tag in the *Positive *group was derived from a single LongSAGE tag, but the tag in both *Negative *and *Either *groups was derived from at least two LongSAGE tags. The maximum number of corresponding LongSAGE tags for a tSAGE tag was 114 for the *Negative *group and 68 for the *Either *group. We also examined the number of UniGene clusters that mapped to each of the 400 significant tSAGE tags. The tSAGE tags in the *Positive *group mapped up to seven UniGene clusters, while the tags in the *Negative *group and *Either *group mapped up to 108 and 66 clusters, respectively. Overall, the significant tSAGE tags in both *Negative *and *Either *groups tend to map to more LongSAGE tags and known genes.

One of the most interesting findings is the analysis of orphan tags. The BLAST  analysis for the 100 randomly selected orphan tags revealed 17 orphan tags with at least 17 bps in the tag completely matched to a gene sequence in human [6]. This frequency (17%) is close to the probability of obtaining one gene sequence perfectly matched to 17 bps of a given tag under an assumed human genome size of 2.864 × 10^9 ^bps (14%) and equal frequency of each nucleotide occurred at a base. The number of matched gene sequences for an orphan tag increases as the number of matched bps decreases (Table 3). A total of 39 gene sequences were identified through this approach. Since the tag sequence used in the BLAST analysis consists of four bps (nucleotide position one to four) from the restriction site and 17 bps (nucleotide position five to 21) from the SAGE tag, we also restricted our selection to tags that have at least all 17 bps in the tag region which match to a gene sequence. The reason for this is that sequencing errors are more likely in the restriction sites rather than in the tag region. Under these criteria, the ending position of the matched segment in the tag sequence is always 21 and the starting position needs to be less than or equal to five. We found nine orphan tags that met these criteria (Table 4). Four of nine orphan tags matched to a single human gene sequence – with 21, 20, and 18 matched bps, which are more likely to be the real transcripts for these four orphan tags.

**Table 3:** Results of BLAST analysis for 100 orphan tags.

**Number of matched basepairs**	**No. of orphan tags mapped to human gene sequences**	**Accumulated percentage in novel gene identification**	**No. of clusters identified**
21	2	2%	2
20	1	3%	1
19	1	4%	3
18	8	12%	10
17	5	17%	23
≥ 17*	9	9%	17

Summary of the number of orphan tags by the number of basepairs matched to a human gene sequence.

*No. of tags have all 17 bps in the tag region matched to a human gene sequence.

**Table 4:** A list of genes mapping to nine orphan tags.

**LongSAGE Tag**	**Start and end of matched location**	**No. of matched basepairs**	**Gene ID**	**Description of the sequence matched to the tag**
**AAATATCCAGAATAGGC**	**2–21**	**20**	**XM_378567.1**	**PREDICTED: Homo sapiens hypothetical LOC400505 (LOC400505), mRNA**

CCAGCCGGGGTGACAGA	5–21	17	NM_000791.3	Homo sapiens dihydrofolate reductase (DHFR), mRNA
CCAGCCGGGGTGACAGA	5–21	17	NM_001874.3	Homo sapiens carboxypeptidase M (CPM), transcript variant 1, mRNA
CCAGCCGGGGTGACAGA	5–21	17	NM_024080.3	Homo sapiens transient receptor potential cation channel, subfamily M, member 8 (TRPM8), mRNA

CCAGTCTGGGCAACAAG	5–21	17	NM_017437.1	Homo sapiens cleavage and polyadenylation specific factor 2, 100 kDa (CPSF2), mRNA
CCAGTCTGGGCAACAAG	5–21	17	NM_181776.1	Homo sapiens solute carrier family 36 (proton/amino acid symporter), member 2 (SLC36A2), mRNA

CCTGGCACTTTGGGAGG	5–21	17	NM_000641.2	Homo sapiens interleukin 11 (IL11), mRNA
CCTGGCACTTTGGGAGG	5–21	17	NM_000997.3	Homo sapiens ribosomal protein L37 (RPL37), mRNA
CCTGGCACTTTGGGAGG	5–21	17	NM_001009899.1	Homo sapiens KIAA2018 (KIAA2018), mRNA
CCTGGCACTTTGGGAGG	5–21	17	NM_001032999.1	Homo sapiens core-binding factor, runt domain, alpha subunit 2; translocated to, 2 (CBFA2T2), transcript variant 3, mRNA
CCTGGCACTTTGGGAGG	5–21	17	NM_001033564.1	Homo sapiens hypothetical protein LOC619208 (LOC619208), mRNA
CCTGGCACTTTGGGAGG	5–21	17	NM_007042.1	Homo sapiens ribonuclease P 14 kDa subunit (RPP14), mRNA
CCTGGCACTTTGGGAGG	5–21	17	NM_030579.1	Homo sapiens outer mitochondrial membrane cytochrome b5 (CYB5-M), mRNA
CCTGGCACTTTGGGAGG	5–21	17	XM_371492.2	PREDICTED: Homo sapiens similar to signal-transducing adaptor protein-2; brk kinase substrate (LOC388949), mRNA
CCTGGCACTTTGGGAGG	5–21	17	XM_379458.2	PREDICTED: Homo sapiens hypothetical LOC401287 (LOC401287), mRNA
CCTGGCACTTTGGGAGG	5–21	17	XM_499056.1	PREDICTED: Homo sapiens hypothetical gene supported by AK127523 (LOC441190), mRNA
CCTGGCACTTTGGGAGG	5–21	17	XM_499503.1	PREDICTED: Homo sapiens hypothetical gene supported by AK127523 (LOC442499), mRNA

**GACTGGAGGTGTGGGGA**	**4–21**	**18**	**NM_014636.1**	**Homo sapiens Ral GEF with PH domain and SH3 binding motif 1 (RALGPS1), mRNA**

**GGTGAGTGGGACCCAGG**	**1–21**	**21**	**NM_012289.3**	**Homo sapiens kelch-like ECH-associated protein 1 (KEAP1), transcript variant 2, mRNA**

GTGCTGGGATAACTGGC	4–21	18	XM_499182.1	PREDICTED: Homo sapiens hypothetical gene supported by AK128305
GTGCTGGGATAACTGGC	5–21	17	NM_005431.1	(LOC441501), mRNA Homo sapiens X-ray repair complementing defective repair in Chinese hamster cells 2 (XRCC2), mRNA
GTGCTGGGATAACTGGC	5–21	17	NM_016094.2	Homo sapiens COMM domain containing 2 (COMMD2), mRNA
GTGCTGGGATAACTGGC	5–21	17	XM_374973.1	PREDICTED: Homo sapiens similar to hypothetical protein (L1H 3 region) – human (LOC400025), mRNA

**GTGGGTCCTCGGGTTGG**	**1–21**	**21**	**NM_194286.2**	**Homo sapiens KIAA1853 protein (KIAA1853), mRNA**

TGGTACACACCTGTAGT	4–21	18	NM_001008528.1	Homo sapiens matrix-remodelling associated 7 (MXRA7), transcript variant 1, mRNA
TGGTACACACCTGTAGT	4–21	18	NM_152920.1	Homo sapiens egf-like module containing, mucin-like, hormone receptor-like 2 (EMR2), transcript variant 6, mRNA
TGGTACACACCTGTAGT	5–21	17	NM_014573.1	Homo sapiens hypothetical protein MAC30 (MAC30), mRNA

Summary of the nine orphan tags that have complete 17 basepairs (bps) in the tag region matched to gene sequences in human. The input sequences for BLAST were 21 bp long including four bps of restriction site. The starting position of the first nucleotide of the LongSAGE tag is the fifth position of the input sequence.

## Discussion

The use of SAGE libraries has been advocated, but technical complexity has limited their use. In addition, the value of long vs. short tag SAGE has not been widely explored. A few facts for a SAGE study are listed below. First, the tSAGE libraries share similar numbers of unique tags and tag counts with the "real" ShortSAGE libraries. The small differences between tSAGE and ShortSAGE libraries may be simply due to the variation between samples. These outcomes imply one advantage for the LongSAGE libraries as they can be analyzed in two ways (as long or short tags). Second, to reach a similar number of total tag counts, LongSAGE libraries, due to greater tag length, need to sequence more clones than ShortSAGE libraries, resulting in increased time and cost. Third, a large number of orphan tags exist in both LongSAGE and ShortSAGE libraries. In fact, LongSAGE libraries have more orphan tags than ShortSAGE libraries – due to their greater specificity in gene mapping.

Identifying differentially expressed genes between tissue samples is often the goal in conducting expression studies. Conclusions on what constitutes a significant change in gene expression are usually guided by the p-values derived from statistical tests. One important feature of our study is our investigation on the potential and serious problem of identifying wrong genes using SAGE libraries, especially ShortSAGE libraries. Are genes or ESTs corresponding to a significant differentially expressed tag real? By utilizing both LongSAGE and tSAGE libraries, we showed that likely only 156 out of 400 significant tSAGE tags (39%) are the presumed true significant tags, because they were derived from significant LongSAGE tags. On the other hand, the 79 significant tSAGE tags in the *Negative *group are probably not truly differentially expressed, because none of their corresponding LongSAGE tags are significant. Since the tag count for a tSAGE tag is the sum of tag counts from its corresponding LongSAGE tags, a false positive result of a tSAGE tag may simply be due to its mapping to multiple LongSAGE tags. In a real setting, this problem will exist for a tag that maps to multiple genes or ESTs. When there are only ShortSAGE data available, we will not be able to dissect the tag-gene relationship as described here. We may make a wrong decision by concluding a significant short SAGE tag by simply looking at the p-value, even if the p-values are very small.

Since all 156 tSAGE tags in the *Positive *group (the presumed true significant tSAGE tags) map to a single LongSAGE tag that has high specificity in tag-to-gene mapping, one potential solution is to take into account the number of UniGene clusters mapped to a tag in the decision making process. Among the 156 tSAGE tags in the *Positive *group (the presumed true significant ones), 67% of tags match to two UniGene clusters. On the other hand, 53% of tSAGE tags in the *Negative *group (the false ones) mapped to more than two UniGene clusters. If we treat the tags that map to two or fewer UniGene clusters as the presumed true significant tags, we will only include 47% of false ones, which is better than including all tags with false positive results.

Through this paper, our tag-to-gene mapping analysis relies on the UniGene database. However, a UniGene cluster does not always imply a gene. It is possible that multiple UniGene clusters refer to the same gene. In our LongSAGE tags analysis, we found that 97.3% of LongSAGE tags are either orphan tags or mapped to a single UniGene Cluster, which is less likely to produce ambiguity of tag-to-gene mapping. For the remaining 2.7% of LongSAGE tags, 1.9% (1044 tags) map to two UniGene clusters. While it is not our main focus to dissect the property of each UniGene cluster in this paper, we found that 10.7% of 1044 LongSAGE tags have the same description for the two clusters even though their UniGene IDs are different. Therefore, it is possible that some of these LongSAGE tags are in fact mapping to a single gene, which may increase the specificity of tag-to-gene mapping for LongSAGE tags.

The large number of orphan tags also represents the limitation of the UniGene database. We showed that there is a potential to use long SAGE tags to identify novel genes that are not listed in the UniGene database. Unlike the short SAGE tag, the long SAGE tag has a sufficient number of nucleotides – allowing us to perform BLAST analysis to search for novel genes. In this study, our criteria in BLAST analysis is to search for at least 17 bps of a SAGE tag matched to a human gene sequence without any gaps. Under this search, we were able to identify 39 genes for 17 orphan tags. More specifically, nine orphan tags were found to have the full 17 bps within the tag region, matching to a human gene sequence, and the number of genes identified reduced to 17. The best results in our BLAST search are the four orphan tags that matched to a single gene sequence by 21, 20, and 18 bps. Considering the probability of obtaining one matched gene sequence is as low as 0.07% for 21 bps, 0.3% for 20 bps, and 4% for 18 bps for a genome size of 2.864 × 10^9 ^bps, it is highly possible that these are real genes corresponding to these four orphan tags. From this BLAST study, we should be able to resolve 4–9% of orphan tags. Although we surveyed only 100 orphan tags, these results are encouraging because we will potentially be able to expand the number of known genes using the LongSAGE library.

Although our SAGE libraries cannot represent other SAGE studies, it provides a good example that one can filter significant tags based on the number of their corresponding genes. In general, it would seem reasonable to use, at most, two corresponding genes as a cutoff to filter significant ShortSAGE tags. Further, if a project aims to be more exclusive in the process of gene selection, one can use the most conservative approach to exclude all significant tags that map to more than one gene.

## Conclusion

The LongSAGE exhibits advantages over ShortSAGE libraries in several aspects. LongSAGE tags appear to have higher specificity in gene mapping than ShortSAGE tags. LongSAGE tags show an advantage over ShortSAGE in identifying novel genes by BLAST analysis, which may help to reduce the number of orphan tags. Most importantly, LongSAGE libraries have advantages in identifying genes that are truly expressed differently between samples, compared to ShortSAGE libraries. In addition we will still be able to perform analysis based on ShortSAGE tags using LongSAGE libraries. This makes the extra costs and experimental time that a LongSAGE library needs worthwhile.

## Methods

### Human brain samples and pathological assessment

Human brain tissues were collected in the Kathleen Price Bryan Brain Bank at the Duke University Alzheimer Disease Research Center (ADRC) and in the Brain Bank of the Center for Human Genetics (CHG) at Duke University Medical Center (DUMC), following the rapid autopsy protocol [7]. The hippocampus was dissected at the time of autopsy, and matching 100–200 mg portions of CA 1–4 were removed and used for RNA isolation and expression studies. Four brain tissue samples, including two AD (Sample IDs: 470 and 589) and two controls (sample IDs: 673 and 707), used in this study were previously described in Xu et al [8]. All four samples have the same apolipoprotein E 3/3 genotype (APOE3/3). The pathological diagnosis of AD was established according to CERAD criteria [9], and the degree of AD pathological changes was staged according to Braak [10]. The AD patients used in this study have pathological changes at the Braak and Braak stage IV and V (B&B stage IV and V), and the control was cognitively and pathologically normal with B&B stage I. Post-mortem delay times ranged from 1:10 to 4:15 hours [8].

### RNA isolation for SAGE library construction

Total RNA was isolated from frozen hippocampus samples of AD patients and controls using TRIzol reagent (Invitrogen) according to the manufacturer's instructions. Briefly, brain tissue was homogenized in TRIzol reagent by Dounce homogenization and the homogenized samples were incubated for five minutes at room temperature. After the addition of chloroform, the mixture was centrifuged to separate the RNA containing aqueous phase from the TRIzol reagent. The aqueous phase was transferred to a fresh tube and the RNA precipitated after adding 0.5 volume of isopropyl alcohol. The RNA pellet was washed once with 75% ethanol, dried, and resuspended in DEPC treated water and stored at -80°C.

### Construction of human hippocampus SAGE libraries

For ShortSAGE library construction, standard protocols as described by Velculescu et al [1], and Basrai and Hieter [11] were used with minor modifications. Briefly, SAGE was performed with 10 μg total RNA isolated from human brain hippocampus samples as outlined above. The cDNA was prepared using the SuperscriptII cDNA synthesis kit (Invitrogen) with gel-purified 5'-biotinylated Oligo(dT)_18 _(Integrated DNA Technologies, Coralville, IA), according to the manufacturer's protocol. *Nla*III and *Bsm*FI restriction enzymes (New England Biolab, Beverly, MA) were used for tag generation. *Bsm*FI digestion was performed at 37°C for 2.5 h (instead of 65°C) using 40 units *Bsm*FI in a 300 μl reaction volume with supplied buffer. After a three-hour concatemerization step, the concatemers were heated at 65°C for 10 minutes, followed by two minutes on ice to enhance cloning efficiency. Purified concatemers were subsequently cloned in the *Sph*I site of pZero-1 (Invitrogen) and transformed in competent ElectroMax DH10B cells (Invitrogen) using a 0.1 cm cuvette and the Gene Pulser II (BioRad). Individual SAGE library clones were selected and PCR amplified using 96-well format Qiagen Real minipreps, and sequenced with ABI 3700 capillary sequencer using BigDye chemistry.

LongSAGE library construction was performed with 10 μg total RNA using the standard SAGE protocol with the modifications according to Saha, et al. [12]. We used the *MmeI *type IIS restriction endonuclease (New England Biolab) to release the linker tag molecules from the cDNA.

### SAGE tag extraction

ShortSAGE tags (10 bps) were extracted from the PHD files with eSAGE software, using a threshold value of PHRED 20 for each base (Margulies and Innis 2000). The SAGE tags were compared between the ShortSAGE AD (S_AD) and ShortSAGE control (S_Ctrl) library using eSAGE software to form a compared ShortSAGE database. LongSAGE tags (17 bps) were extracted from raw sequence data of LongSAGE libraries using SAGE2000 version 4.5 Analysis Software. We directly merged the SAGE tags from the LongSAGE AD (L_AD) and LongSAGE control (L_Ctrl) libraries to generate a compared LongSAGE database. Both compared ShortSAGE and LongSAGE databases were mapped to UniGene build 182 (National Center for Biotechnology Information, NCBI).

### SAGE data analysis

In addition to the four SAGE libraries described above, we used the same strategy employed by Lu et al. [5] to generate two additional short SAGE libraries based on the LongSAGE libraries. We truncated the seven 5' bps of each long SAGE tag to generate truncated LongSAGE (tSAGE) library, which is analogous to the ShortSAGE library – as each tSAGE tag has only 10 bps. The tag count of a tSAGE tag is the sum of tag counts of LongSAGE tags that have the same first 10 bps. Hereafter, we refer to the two tSAGE libraries as T_AD for the tSAGE AD library and T_Ctrl for the tSAGE control library. Similarly, we generated and compared a SAGE database for T_AD and T_Ctrl, and mapped tSAGE tags to UniGene build 182. This allows us to directly compare results for long and short SAGE (i.e. LongSAGE and tSAGE) tags derived from the same tissue samples. We utilized these six libraries (three compared SAGE databases) to investigate the information content of long and short SAGE libraries.

First, the data was summarized for these six SAGE libraries. We computed the number of unique tags, the total tag counts, the number of tags that map to UniGene, and the number of tags with no UniGene information (i.e. the orphan tags) for each library. We also evaluated the specificity of the long and short SAGE tags for gene mapping. We computed the number of genes corresponding to each tag for the three compared SAGE datasets. To estimate the percentage of redundant short SAGE tags that can be resolved by the long SAGE tags, we mimicked the approach of Lu et al. [5] using the LongSAGE and tSAGE libraries. We obtained a set of unique LongSAGE tags with tag counts greater than one. Then, we computed the numbers of unique and redundant tSAGE tags that correspond to these LongSAGE tags. In other words, these redundant tSAGE tags can be resulted if their corresponding LongSAGE tags are known. Further, we investigated the tag-to-gene mapping pattern of the tSAGE tags that originally map to a single UniGene cluster under the LongSAGE tag format.

Second, we examined the performance of the LongSAGE, tSAGE, and ShortSAGE libraries in identifying differentially expressed genes. Chi-square and Fisher exact tests, as previously described [13], were used to test differences in expression levels between AD and control for each tag in each compared SAGE dataset. Since it is not our goal to provide a set of candidate genes, but rather use the results to compare the relationship between significant short and long SAGE tags, we applied a nominal significance level of 0.05 to declare significant tags without considering a correction for multiple testing. We summarized the number of significant tags for each compared SAGE dataset. For all significant tSAGE tags, we investigated the number of its corresponding long tags. We compared the LongSAGE tag counts per tSAGE tag among three groups.

Finally, UniGene serves as a database to interpret the SAGE tags. Each UniGene cluster contains sequences that represent a unique gene or EST. Since the UniGene set is based on expressed mRNAs, it represents only a small portion of the genome. Although there are more than 53,000 unique UniGene entries, a large number of orphan tags are still found in both ShortSAGE and LongSAGE libraries. Here, we investigate whether LongSAGE tags can help us identify genes corresponding to these orphan tags and whether they represent real genes or are artifacts of library construction and analysis.

Since the maximum length of a LongSAGE tag is up to 21 bps (including the cut site), it is possible to search genes corresponding to these long tags using sequencing alignment tools, such as BLAST. BLAST finds regions of local similarity between DNA sequences. Under the assumption of equal probability of sampling a nucleotide at each base, the probability of obtaining an exact matched sequence with *k *bps is (*1/4*)^*k*^. Assuming that the human genome consists with *N *bps of nucleotides, the approximated probability of obtaining one matched chromosomal segment with k bps is

(N−k+1)(14)k(1−(14)k)N−k,
 MathType@MTEF@5@5@+=feaafiart1ev1aaatCvAUfKttLearuWrP9MDH5MBPbIqV92AaeXatLxBI9gBaebbnrfifHhDYfgasaacH8akY=wiFfYdH8Gipec8Eeeu0xXdbba9frFj0=OqFfea0dXdd9vqai=hGuQ8kuc9pgc9s8qqaq=dirpe0xb9q8qiLsFr0=vr0=vr0dc8meaabaqaciaacaGaaeqabaqabeGadaaakeaacqGGOaakcqWGobGtcqGHsislcqWGRbWAcqGHRaWkcqaIXaqmcqGGPaqkcqGGOaakdaWcaaqaaiabigdaXaqaaiabisda0aaacqGGPaqkdaahaaWcbeqaaiabdUgaRbaakiabcIcaOiabigdaXiabgkHiTiabcIcaOmaalaaabaGaeGymaedabaGaeGinaqdaaiabcMcaPmaaCaaaleqabaGaem4AaSgaaOGaeiykaKYaaWbaaSqabeaacqWGobGtcqGHsislcqWGRbWAaaGccqGGSaalaaa@4634@

if all chromosomal segments of k bps are independent, and the expected number of chromosomal segments that match to a tag with *k *bps is (*N-k+1*)(*1/4*)^*k*^. The number of genes matching to a given tag decreases as the number of required matched bps (*k*) in the tag increases. If we assume that the human genome consists with 2.864 × 10^9 ^bps of nucleotides (Goden path length at ), we may expect to find 10 sequence segments matched to a 14-bp tag sequence. This number reduces to less than one when we require the number of bps to match to a tag to be 16+ bps. Clearly, a larger *k *will have a higher accuracy in gene identification than a smaller *k*. Based on the above calculations; we used 17+ bps as our search criteria in BLAST analysis. However, this computation did not take into account some genes that may be highly homologous to each other. Here, we examine the frequencies of obtaining perfect matched gene sequences for orphan tags through BLAST analysis. A gene sequence is considered a perfect match with an orphan tag if a gene sequence has a segment matched to a complete portion of a tag, that is, no gaps (unmatched nucleotides) within the sequence are allowed. We randomly selected 100 orphan LongSAGE tags from the L_Ctrl library and screen the 21 bps LongSAGE tag sequences by BLAST. We selected the tags that show a perfect match to human genes with at least 17 bps.

## Authors' contributions

YJL supervised statistical analysis, drafted and revised the manuscript, and is responsible for the content of the paper. PX generated SAGE libraries used in this study and also helped manuscript preparation. XQ performed the data analysis. DES involved in patient ascertainment. CMH involved in autopsy works. JLH and MAP are the PIs of Alzheimer studies and grants which funded part of the research. They provided samples available for this study. JRG supervised molecular biology components and helped manuscript preparation.

## References

[B1] Velculescu VE, Zhang L, Vogelstein B, Kinzler KW (1995). Serial analysis of gene expression. Science.

[B2] van Ruissen F, Ruijter JM, Schaaf GJ, Asgharnegad L, Zwijnenburg DA, Kool M, Baas F (2005). Evaluation of the similarity of gene expression data estimated with SAGE and Affymetrix GeneChips. BMC Genomics.

[B3] Lash AE, Tolstoshev CM, Wagner L, Schuler GD, Strausberg RL, Riggins GJ, Altschul SF (2000). SAGEmap: a public gene expression resource. Genome Res.

[B4] Boon K, Osorio EC, Greenhut SF, Schaefer CF, Shoemaker J, Polyak K, Morin PJ, Buetow KH, Strausberg RL, De Souza SJ, Riggins GJ (2002). An anatomy of normal and malignant gene expression. Proc Natl Acad Sci USA.

[B5] Lu J, Lal A, Merriman B, Nelson S, Riggins G (2004). A comparison of gene expression profiles produced by SAGE, long SAGE, and oligonucleotide chips. Genomics.

[B6] Altschul SF, Madden TL, Schaffer AA, Zhang J, Zhang Z, Miller W, Lipman DJ (1997). Gapped BLAST and PSI-BLAST: a new generation of protein database search programs. Nucleic Acids Res.

[B7] Hulette CM, Welsh-Bohmer KA, Crain B, Szymanski MH, Sinclaire NO, Roses AD (1997). Rapid brain autopsy. The Joseph and Kathleen Bryan Alzheimer's Disease Research Center experience. Arch Pathol Lab Med.

[B8] Xu PT, Li YJ, Qin XJ, Scherzer CR, Xu H, Schmechel DE, Hulette CM, Evin J, Gullans SR, Haines J, Pericak-Vance MA, Gilbert JR (2006). Differences in apolipoprotein E3/3 and E4/4 allele-specific gene expression in hippocampus in Alzheimer disease. Neurobiol Dis.

[B9] Mirra SS, Heyman A, McKeel D, Sumi SM, Crain BJ, Brownlee LM, Vogel FS, Hughes JR, van Belle G, Berg L (1991). The Consortium to Establish a Registry for Alzheimer's Disease (CERAD). Part II. Standardization of the neuropathologic assessment of Alzheimer's disease. Neurology.

[B10] Braak H, Braak E (1991). Neuropathological stageing of Alzheimer-related changes. Acta Neuropathol (Berl).

[B11] Basrai MA, Hieter P (2002). Transcriptome analysis of Saccharomyces cerevisiae using serial analysis of gene expression. Methods Enzymol.

[B12] Saha S, Sparks AB, Rago C, Akmaev V, Wang CJ, Vogelstein B, Kinzler KW, Velculescu VE (2002). Using the transcriptome to annotate the genome. Nat Biotechnol.

[B13] Hauser MA, Li YJ, Takeuchi S, Walters R, Noureddine M, Maready M, Darden T, Hulette C, Martin E, Hauser E, Xu H, Schmechel D, Stenger JE, Dietrich F, Vance J (2003). Genomic convergence: identifying candidate genes for Parkinson's disease by combining serial analysis of gene expression and genetic linkage. Hum Mol Genet.

